# Supplementary material for: Specialized interfaces of Smc5/6 control hinge stability and DNA association
Source: Nat Commun. 2017 Jan 30;8:14011. doi: 10.1038/ncomms14011 (PMC5290277; doi:10.1038/ncomms14011)
Supplement: Supplementary Information — Supplementary Figures, Supplementary Tables and Supplementary References [file ncomms14011-s1.pdf]

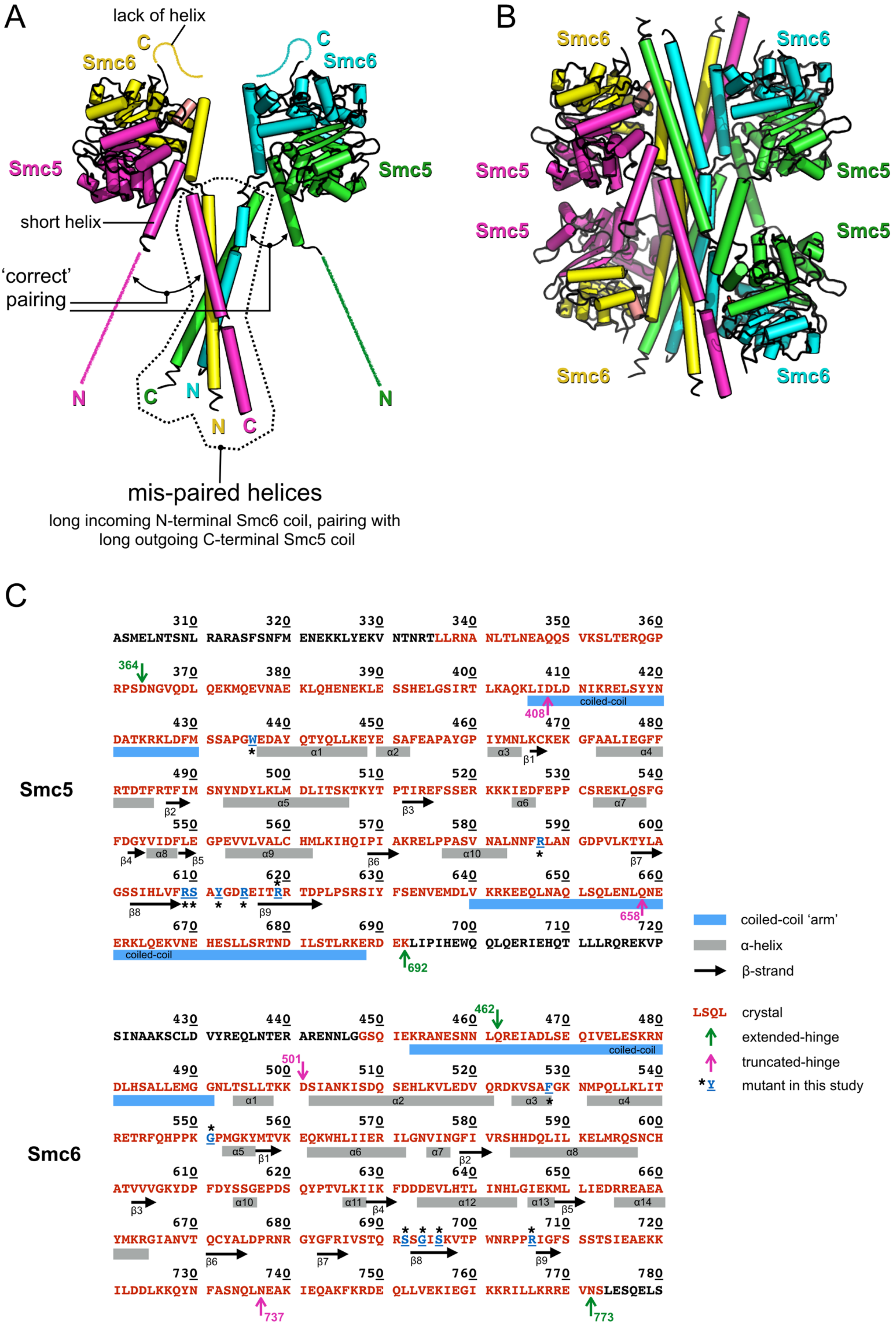

A
lack of helix
C
Smc6
C
Smc6
Smc5
Smc5
short helix
'correct' pairing
N
C
N
N
C
N
mis-paired helices
long incoming N-terminal Smc6 coil, pairing with long outgoing C-terminal Smc5 coil
B
Smc6
Smc6
Smc5
Smc5
Smc5
Smc5
Smc6
Smc6
C
Smc5
310 320 330 340 350 360
ASMELNTSNL RARASFSNFM ENEKKLYEKV NTNRTLLRNA NLTLNEAQQS VKSLTERQGP
364 370 380 390 400 410 420
RPSDNGVQDL QEKMQEVNAE KLQHENEKLE SSHELGSIRT LKAQKLIDLD NIKRELSYYN
408 coiled-coil
430 440 450 460 470 480
DATKRKLDFM SSAPGWEDAY QTYQLLKEYE SAFEAPAYGP IYMNLKCKEK GFAALIEGFF
α1 α2 α3 β1 α4
490 500 510 520 530 540
RTDTFRTFIM SNYNDYLKLM DLITSKTKYT PTIREFSSER KKKIEDFEPP CSREKLQSFG
β2 α5 β3 α6 α7
550 560 570 580 590 600
FDGYVIDFLE GPEVVLVALC HMLKIHQIPI AKRELPPASV NALNNFRLAN GDPVLKTYLA
β4 α8 β5 α9 β6 α10 β7
610 620 630 640 650 660
GSSIHLVFRS AYGDREITRR TDPLPSRSIY FSENVEMDLV KRKEEQLNAQ LSQLENLQNE
β8 β9 658
670 680 690 700 710 720
ERKLQEKVNE HESLLSRTND ILSTLRKERD EKLIPIHEWQ QLQERIEHQT LLLRQREKVP
coiled-coil 692
Smc6
462
430 440 450 460 470 480
SINAAKSCLD VYREQLNTER ARENNLGGSQ IEKRANESNN LQREIADLSE QIVELESKRN
coiled-coil
501
490 500 510 520 530 540
DLHSALLEMG GNLTSLLTKK DSIANKISDQ SEHLKVLEDV QRDKVSAFGK NMPQLLKLIT
α1 α2 α3 α4
550 560 570 580 590 600
RETRFQHPPK GPMGKYMTVK EQKWHLIIER ILGNVINGFI VRSHHDQLIL KELMRQSNCH
α5 β1 α6 α7 β2 α8
610 620 630 640 650 660
ATVVVGKYDP FDYSSGEPDS QYPTVLKIIK FDDDEVLHTL INHLGIEKML LIEDRREAEA
β3 α10 α11 β4 α12 α13 β5 α14
670 680 690 700 710 720
YMKRGIANVT QCYALDPRNR GYGFRIVSTQ RSSGISKVTP WNRPPRIGFS SSTSIEAEKK
β6 β7 β8 β9
730 740 750 760 770 780
ILDDLKKQYN FASNQLNEAK IEQAKFKRDE QLLVEKIEGI KKRILLKRRE VNSLESQELS
737 773
coiled-coil 'arm'
α-helix
β-strand
LSQL crystal
extended-hinge
truncated-hinge
*Y mutant in this study

Supplementary Figure 1

The asymmetric unit of Smc5/6-hinge crystals

(A) The asymmetric unit of Smc5/6-hinge crystals contains two hetero-dimers, shown in molecular cartoon representation. Smc5 is coloured magenta and green, and Smc6 yellow and cyan. The constructs expressing Smc5 (aa 336-692) and Smc6 (448-703) encode mismatched lengths of coiled-coil, at both the N- and C-termini. This has resulting in mis-pairing of the incoming N-terminal coil of Smc6, with the outgoing C-terminal coil of Smc5 in the asymmetric unit. (B) Partially expanded crystal lattice, highlighting the complex arrangement of the component hetero-dimers. (C) Annotated amino acid sequence for the hinge-regions of *S.pombe* Smc5 and Smc6. See associated key for full details.

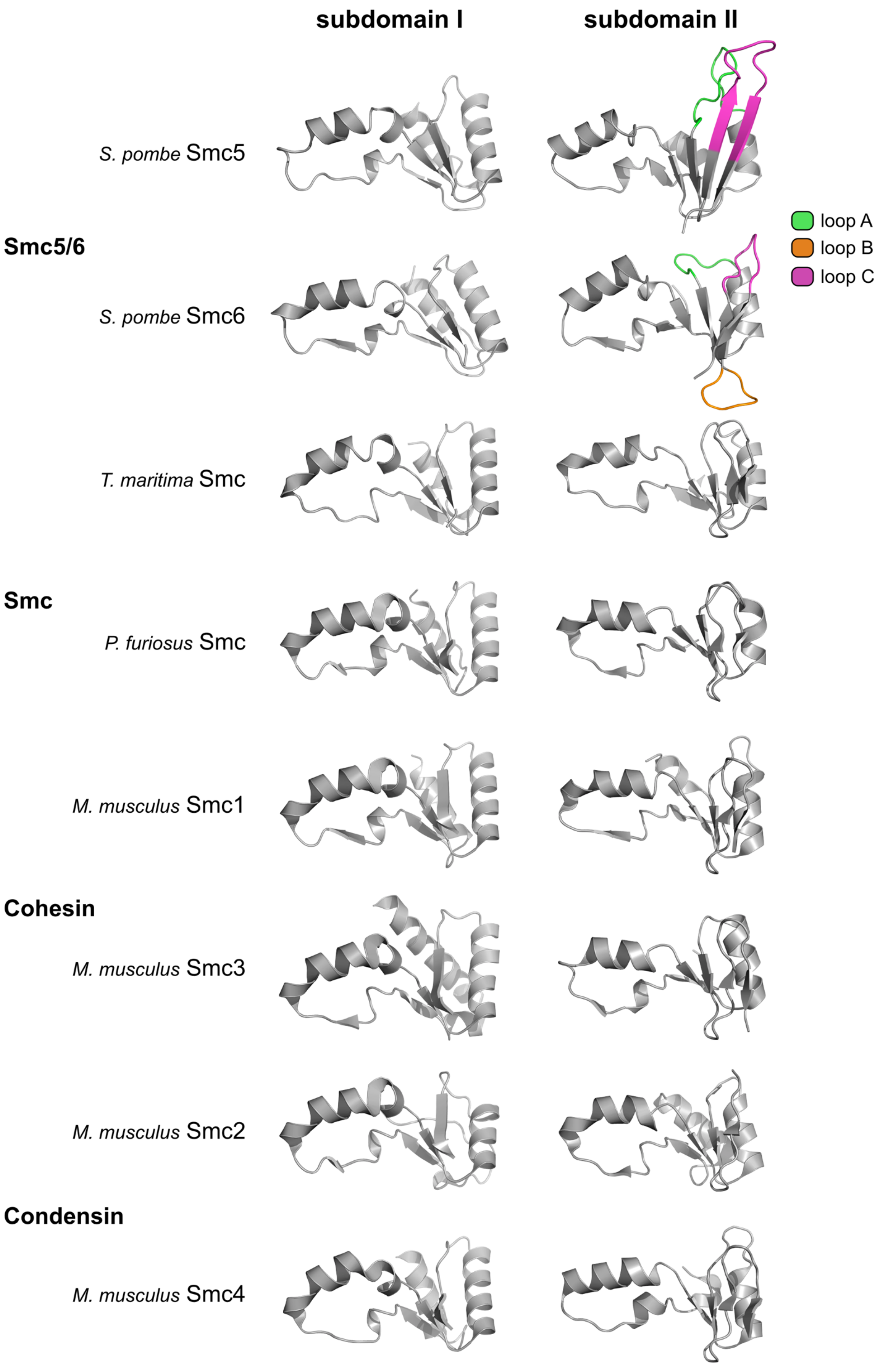
subdomain I
subdomain II
loop A
loop B
loop C
Smc5/6
S. pombe Smc5
S. pombe Smc6
Smc
T. maritima Smc
P. furiosus Smc
Cohesin
M. musculus Smc1
M. musculus Smc3
Condensin
M. musculus Smc2
M. musculus Smc4

Supplementary Figure 2

Subdomains of selected SMC-family proteins

Molecular cartoon representations are show for subdomain I (left) and subdomain II (right) for the indicated SMC proteins. The points of amino acid insertion in subdomain II of Smc5 and Smc6 are additionally highlighted. Loop A, B and C are coloured green, orange and magenta respectively.

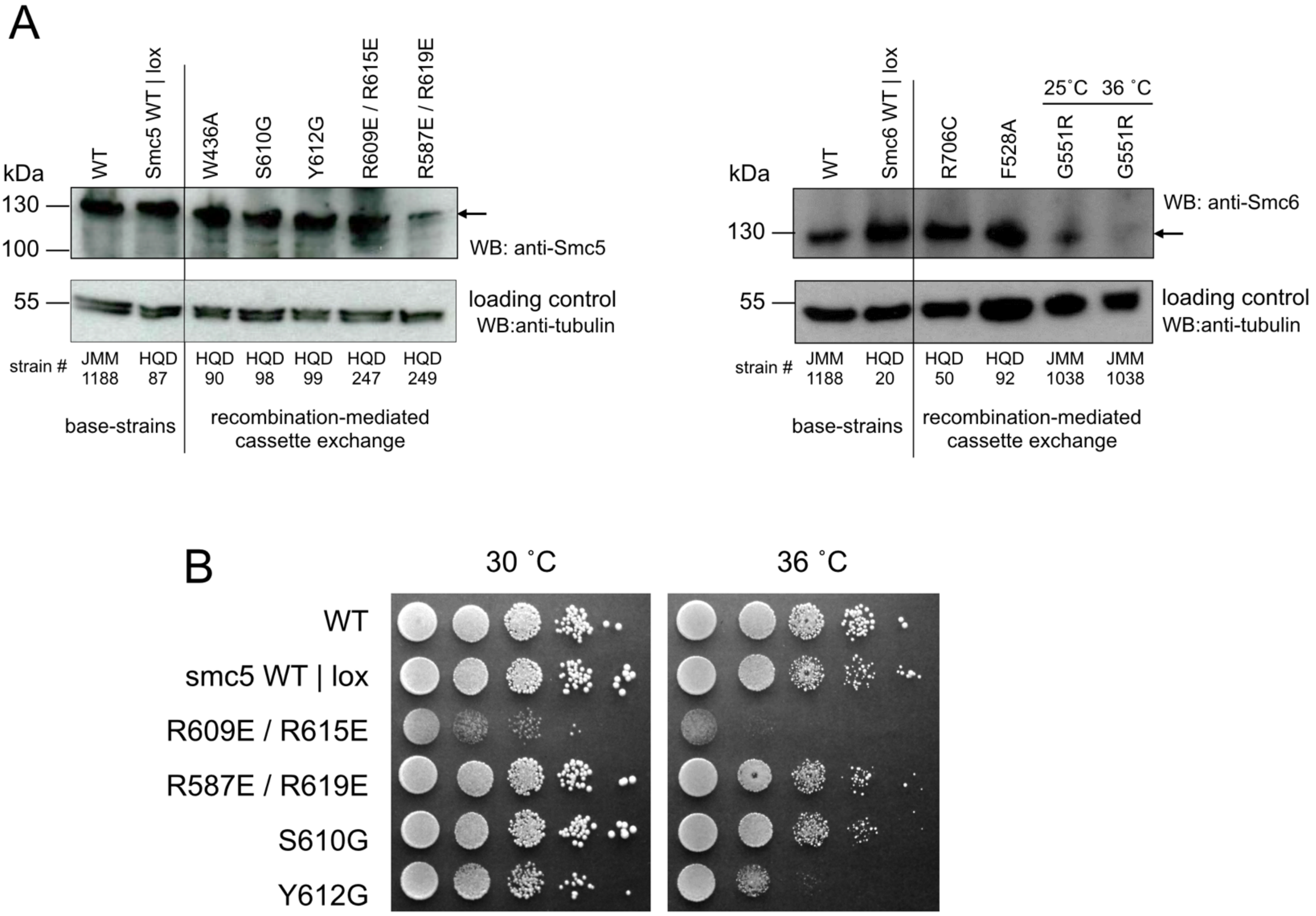

Supplementary Figure 3

Analysis of yeast strains

(A, left) Western blot confirming expression of Smc5 in the indicated *S.pombe* strains. (A, right) Western blot confirming expression of Smc6 in the indicated *S.pombe* strains. The JMM1038 strain, containing the temperature sensitive Smc6 mutation G551R (*smc6-T2*) was incubated at 25 °C or 36 °C for a period of 4 hours prior to harvesting and lysis. The loss of signal at 36 °C confirms specificity of the Smc6 antibody. (B) Temperature sensitivity of Smc5-Loop C and Arginine-pair mutants.

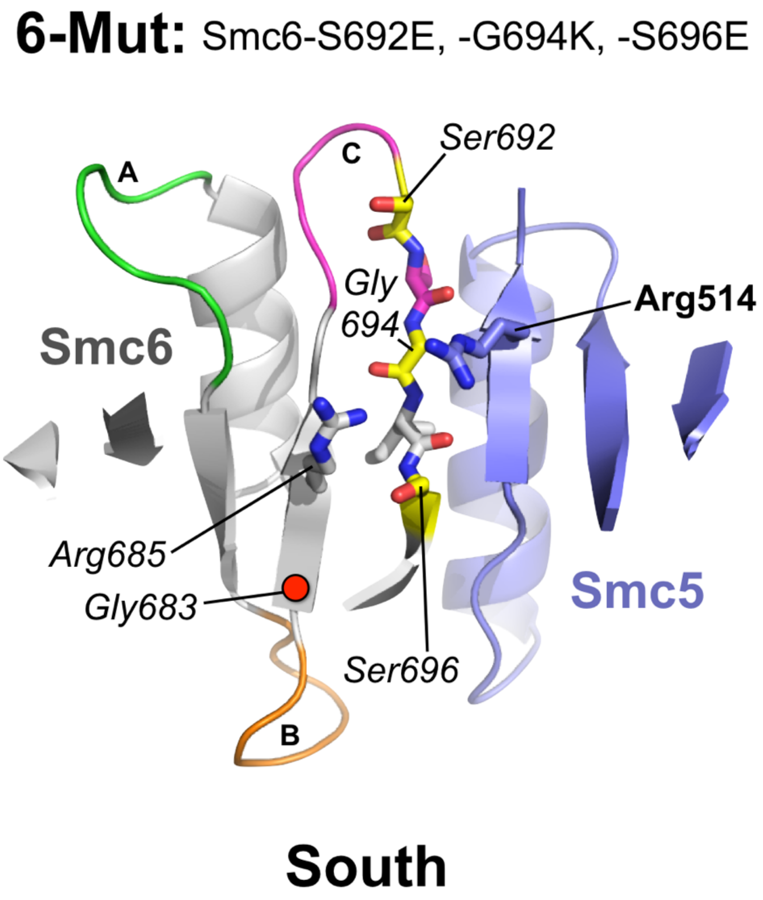

Supplementary Figure 4

Molecular details for the 6-Mut series of mutations

Molecular cartoon representation of the ‘South’ interface, highlighting the position of the amino acids mutated in Smc6, in order to generate ‘6-Mut’ (coloured in yellow).

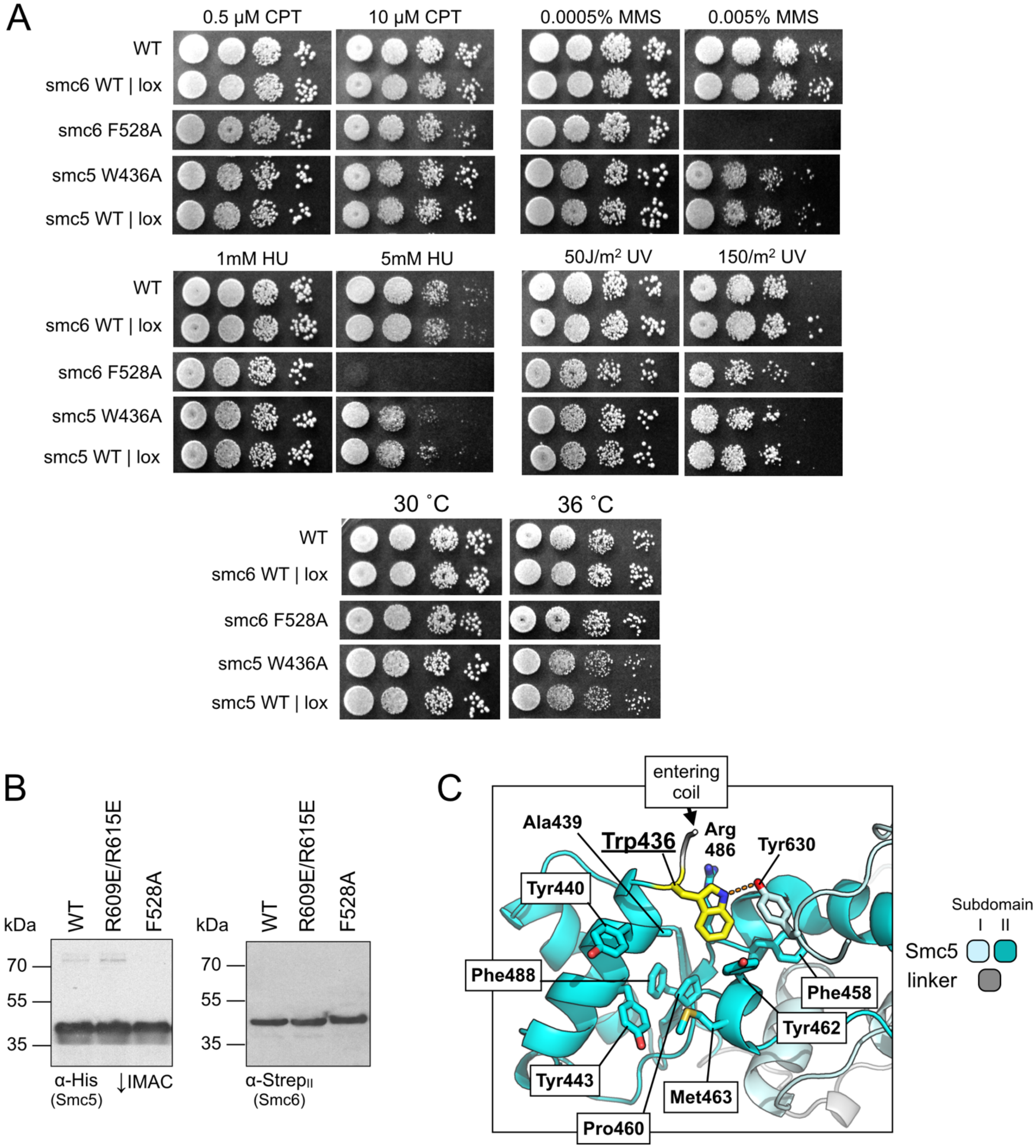
A
0.5 µM CPT
10 µM CPT
0.0005% MMS
0.005% MMS
WT
smc6 WT | lox
smc6 F528A
smc5 W436A
smc5 WT | lox
1mM HU
5mM HU
50J/m² UV
150/m² UV
30 ˚C
36 ˚C
B
kDa
WT
R609E/R615E
F528A
70
55
35
α-His
(Smc5)
↓IMAC
α-StrepII
(Smc6)
C
entering coil
Ala439
Trp436
Arg 486
Tyr630
Tyr440
Phe488
Phe458
Tyr462
Tyr443
Met463
Pro460
Subdomain
I II
Smc5
linker

Supplementary Figure 5

DNA damage and temperature sensitivity of the Smc6-F528A and Smc5-W436A *S.pombe* strains

(A) Doses and type of treatment are as indicated. (B) Co-expression / co-purification assay. His-Tagged Smc5-hinge was co-expressed with StrepII-tagged Smc6-hinge in *E.coli.* After lysis, and clarification, the soluble fraction was passed through a IMAC column, capturing Smc5-hinge. After successive washes, to remove any unbound material, the amount of co-purified Smc6-hinge was assessed by western blot. (C) Molecular details of the Smc5-W436 locus – which is predominantly hydrophobic in nature. Key amino acids residues are labelled. Trp436 is additionally highlighted in yellow.

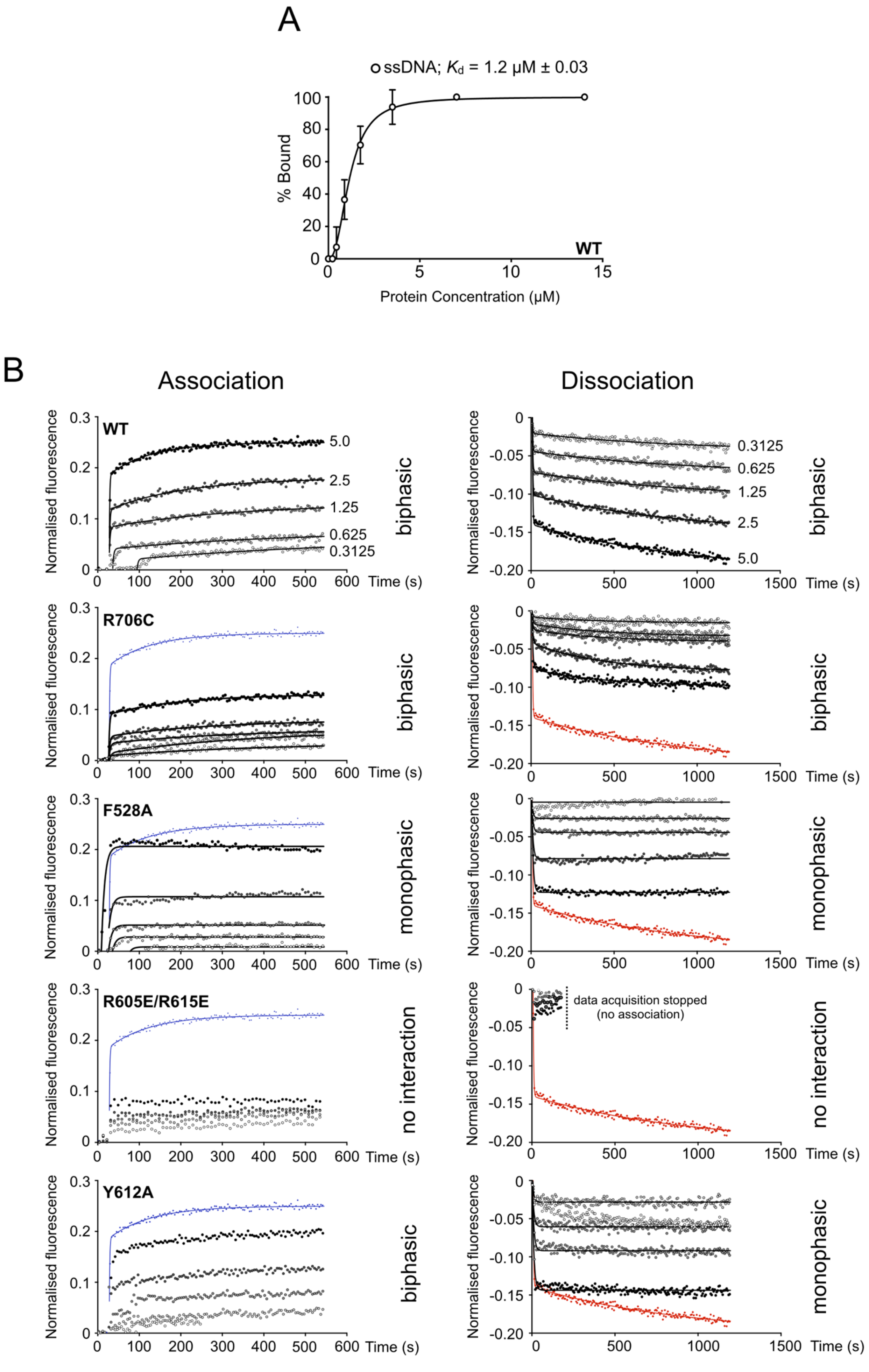

A
ssDNA; Kd = 1.2 µM ± 0.03
% Bound
Protein Concentration (µM)
WT
B
Association
Dissociation
Normalised fluorescence
Time (s)
WT
5.0
2.5
1.25
0.625
0.3125
biphasic
R706C
biphasic
F528A
monophasic
R605E/R615E
no interaction
data acquisition stopped
(no association)
Y612A
biphasic
monophasic

Supplementary Figure 6

Interactions of WT and mutant Smc5/6-hinge with ssDNA

(A) Quantification of EMSA experiment (Figure 6B in manuscript), data-points represent the mean of 3 experiments, with error bars indicating one standard deviation. Dissociation constants (K_d) were determined by least-squares fitting of a one-site model. (B) Representative SwitchSENSE association and dissociation F_{down} sensorgrams for the indicated Smc5/6-hinge protein. For ease of comparison the association (coloured blue) or dissociation curve (red) for the wild-type protein at a concentration of 5 μM is also shown for each mutant.

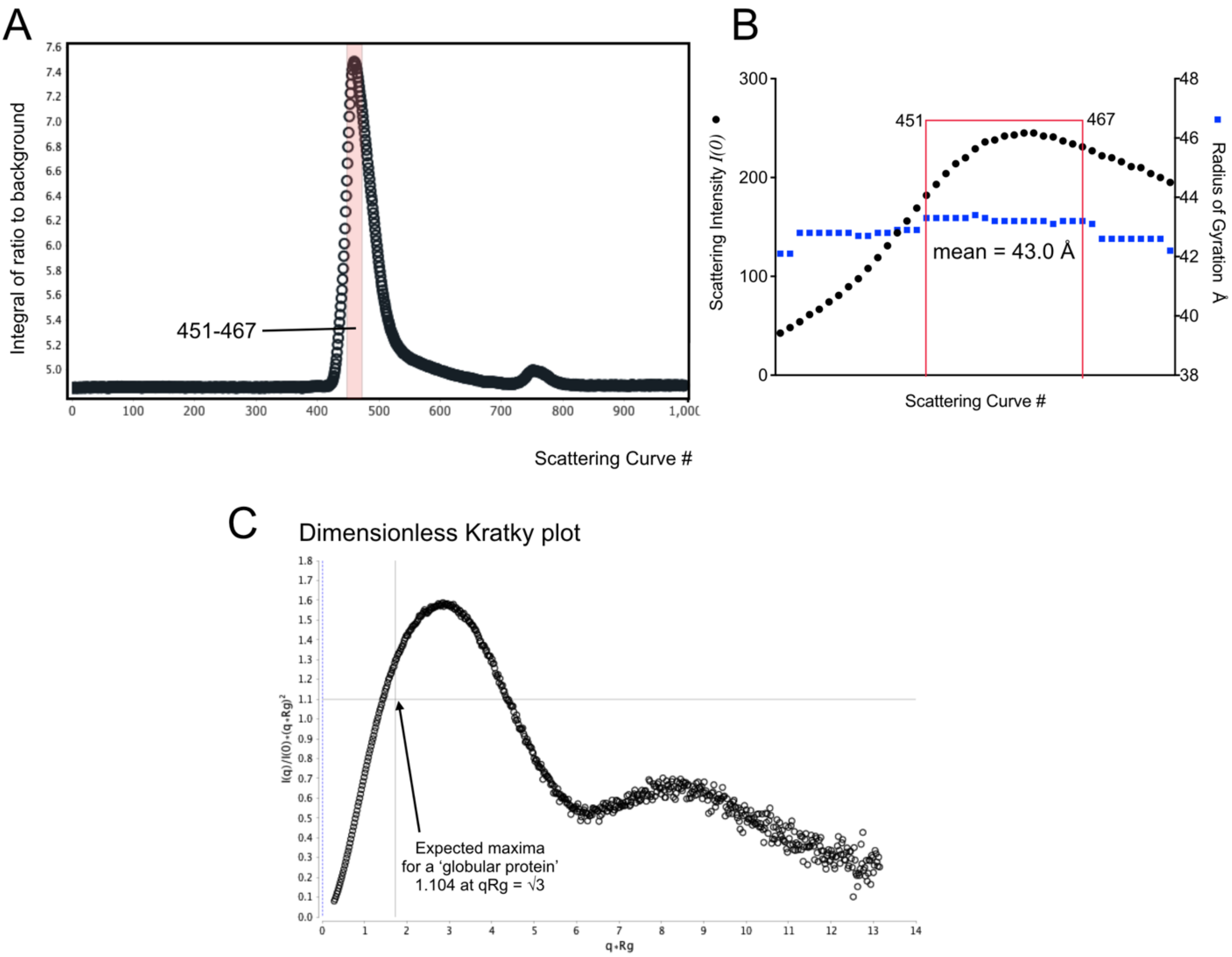

D

	**ATSAS** (2.7.2)	**ScÅtter** (3.0c)
Guinier R_g, Å	44.6 ± 1.5	43.0
Guinier I(0), arbitrary units	234.6 ± 0.3	231
Quality	0.96	-
Aggregated?	0.0049	-
P*(r)* R_g, Å	45.1 ± 0.4	44.7
P*(r)* I(0), arbitrary units	235 ± 0.9	232
Porod volume estimate, $Å^3$	161185	178207
D_{max}, Å	156	165

Supplementary Figure 7

Supporting data for Small-angle X-ray Scattering (SAXS) experiments

(A,B) Scattering profiles corresponding to curves #451-467 (red rectangle) were selected for downstream analysis and averaging; they covered a wide concentration range, yet produced a consistent value for R_g of ~43 Å (ScÅtter[1])

(C) Dimensionless Kratky plot (ScÅtter) for the scattering profile, obtained by merging and averaging curves #451-467. The Smc5/6-hinge does not have a compact, globular structure in solution, it instead adopts an extended conformation with some intrinsic level of flexibility[2].

(D) Summary of parameters determined for Smc5/6-hinge using SAXS with the indicated software packages ATSAS[3] and ScÅtter[1].

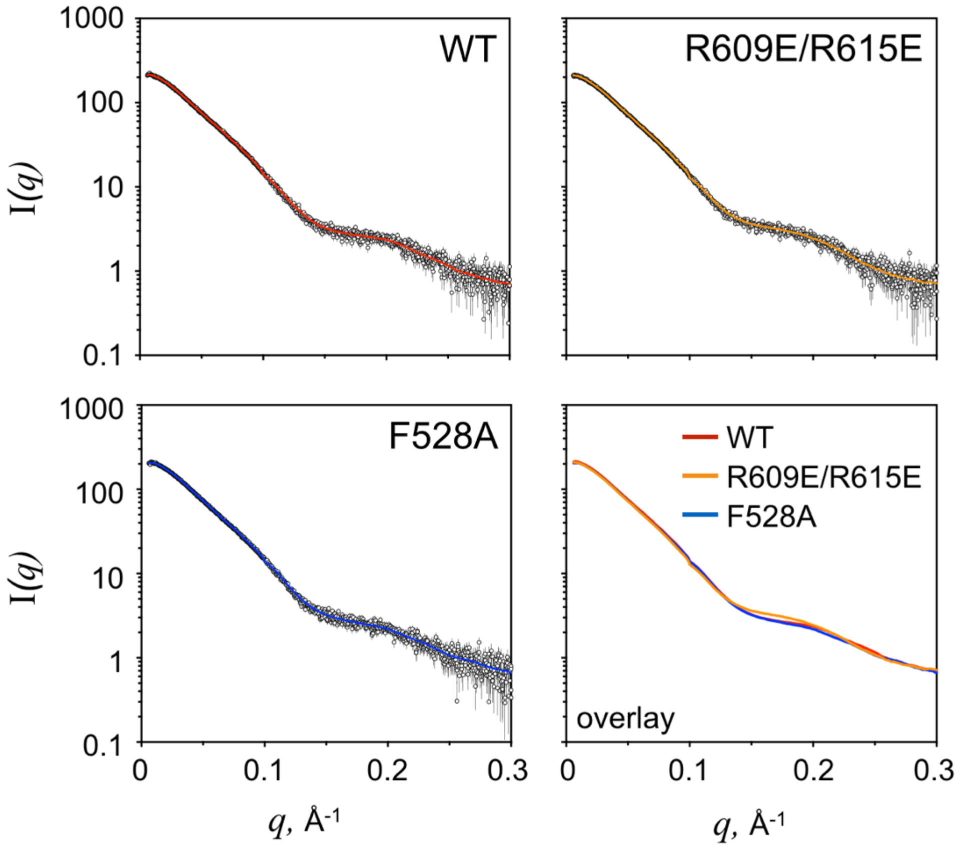

Supplementary Figure 8

Scattering profiles of WT Smc5/6-hinge, R609E/R615E and F528A mutants

Experimental data (open circles) with smoothed polynomial line-of-best-fit are shown in each case.

Figure 3B [reverse orientation]

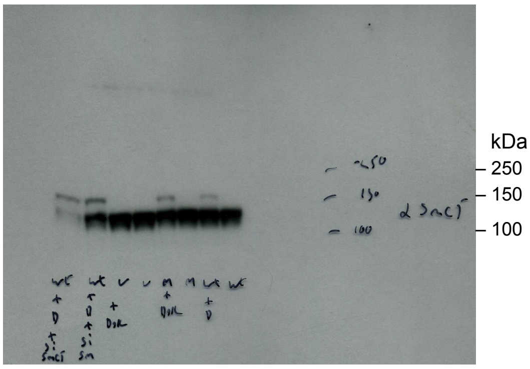

α-Smc5

Figure 3B [reverse orientation]

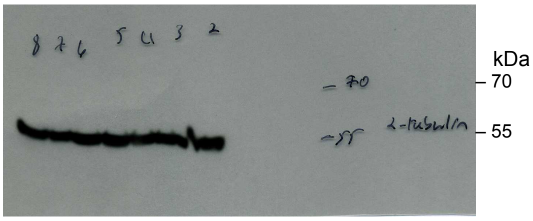

α-tubulin

Figure 4D α-Strep$_{II}$ (Smc6)

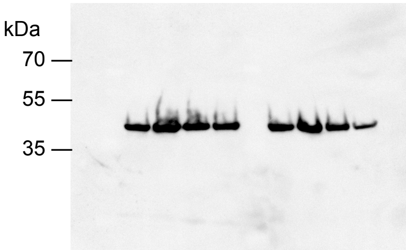

Figure 4D α-His (Smc5)

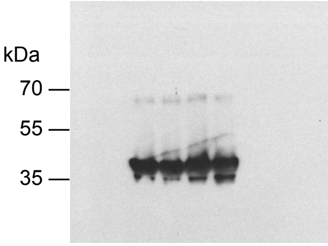

Figure 4D α-Strep$_{II}$ (Smc6)

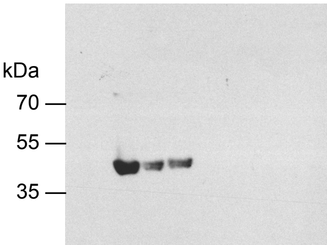

Figure 6B

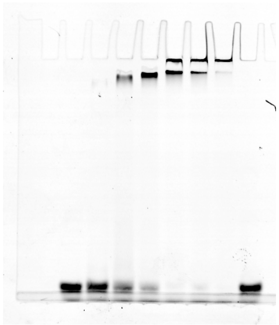

Figure 6B inset

section presented

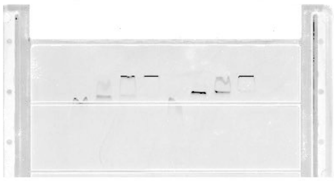

Figure 6B inset

section presented

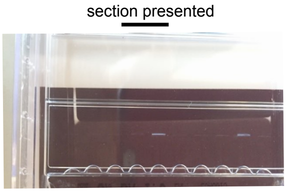

Figure 6C

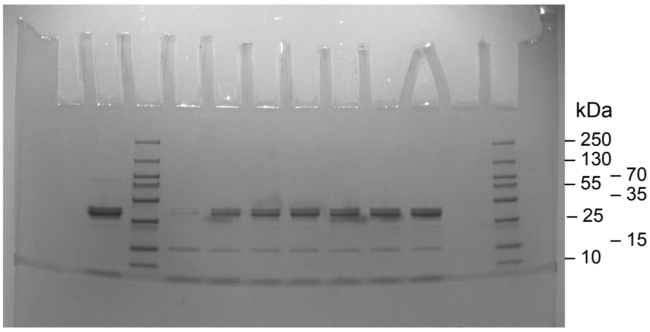

Supplementary Figure 9

Uncropped / uncapped images of gels and western blots

Supplementary Table 1

Amino acid boundaries of expression constructs

	Smc5		Smc6	
	N-terminus	C-terminus	N-terminus	C-terminus
Crystal structure	336	692	448	703
Extended-hinge	364	692	462	773
Truncated-hinge	408	658	501	737

Supplementary Table 2

X-ray diffraction: Data collection, phasing and refinement statistics

	Native	SeMet
**Data collection**		
Space group	$C222_1$	$C222_1$
Cell dimensions		
a, *b*, *c* (Å)	172.56 196.46 122.44	171.36, 195.06, 121.18
α, β, γ (°)	90, 90, 90	90, 90, 90
Wavelength	0.9795	0.9788
Resolution (Å)	45.6 (2.75)	48.8 (4.7)
Mn I / σ*I*	11.9 (1.4)	11.4 (8.9)
Mn I, $CC_{1/2}$	0.998 (0.530)	0.995 (0.983)
Completeness (%)	100 (100)	99.9 (100)
Redundancy	6.6 (6.7)	12.6 (12.9)
**Refinement**		
Resolution (Å)	45.6 (2.75)	
No. unique reflections	54252	
R_{work} / R_{free}	0.21 / 0.25	
No. atoms	8697	
Macromolecules	8575	
Ligands	63	
Solvent	132	
B-factors		
Wilson	62.3	
ADP (mean)		
Macromolecules	82.2	
Ligands	132.8	
Solvent	65.9	
R.m.s deviations		
Bond lengths (Å)	0.007	
Bond angles (°)	0.898	
**Molprobity**		
All atom clashscore	13.84	
**Ramachandran**		
Outliers	0.83%	
Allowed	2.49 %	
Favoured	96.68 %	

*Values in parentheses are for highest-resolution shell.

Supplementary Table 3

Yeast strains

Strain name	Genotype	Notes
JMM1188	h- smt0 ade6-704 leu1-32 ura4-D18	Wild type (WT)
JMM1038	smc6-G551R ade6-704 leu1-32 ura4-D18	Sergeant *et al.*[1]
HQD20	smc6::loxP-smc6-ura4-loxM ade6-704 leu1-32 ura4-D18	This study
HQD50	smc6::loxP-smc6-R706C-loxM ade6-704 leu1-32, ura4-D18	This study
HQD87	smc5::loxP-5'-UTR-smc5-ura4-loxM ade6-704 leu1-32 ura4-D18	This study
HQD90	smc5::loxP-5'-UTR-smc5-W436A-loxM ade6-704 leu1-32 ura4-D18	This study
HQD92	smc6::loxP-Smc6-F528A-loxM ade6-704 leu1-32 ura4-D18	This study
HQD98	smc5::loxP-5'-UTR-smc5-S610G-loxM ade6-704 leu1-32 ura4-D18	This study
HQD99	smc5::loxP-5'-UTR-smc5-Y612G-loxM ade6-704, leu1-32 ura4-D18	This study
HQD247	smc5::loxP-smc5-R609E R615E-loxM, ade6-704 leu1-32 ura4-D18	This study
HQD249	smc5::loxP-smc5-R587E R619E-loxM ade6-704 leu1-32 ura4-D18	This study
YMS83	smc5+/smc5::loxP-5'-UTR-smc5-ura4-loxM ade6-M210/ade6-M216 leu1-32/leu1-32 ura4-D18ura4-d18 h+/h- smt0	This study
YMS99	smc5+/smc5::loxP-5'-UTR- smc5-S610G Y612G-loxM ade6-M210/ade6-M216 leu1-32/leu1-32 ura4-D18ura4-d18 h+/h- smt0	This study

Supplementary References

1. Forster, S., Apostol, L. & Bras, W. Scatter: software for the analysis of nano- and mesoscale small-angle scattering. *Journal of Applied Crystallography* **43**, 639-646 (2010).
2. Durand, D. et al. NADPH oxidase activator p67(phox) behaves in solution as a multidomain protein with semi-flexible linkers. *J Struct Biol* **169**, 45-53 (2010).
3. Petoukhov, M.V. et al. New developments in the ATSAS program package for small-angle scattering data analysis. *Journal of Applied Crystallography* **45**, 342-350 (2012).
